# Percutaneous Rotational Mechanical Atherectomy Plus Thrombectomy Using Rotarex S Device in Patients With Acute and Subacute Lower Limb Ischemia: A Review of Safety, Efficacy, and Outcomes

**DOI:** 10.3389/fcvm.2020.557420

**Published:** 2020-10-22

**Authors:** Romaric Loffroy, Nicolas Falvo, Christophe Galland, Léo Fréchier, Frédérik Ledan, Marco Midulla, Olivier Chevallier

**Affiliations:** ImViA Laboratory-EA 7535, Department of Vascular and Interventional Radiology, Image-Guided Therapy Center, François-Mitterrand University Hospital, Dijon, France

**Keywords:** mechanical thrombectomy, peripheral arterial occlusion, percutaneous transluminal angioplasty, stent placement, atherectomy

## Abstract

Acute and subacute ischemia of lower limbs is associated with high risk of amputation and potential severe life-threatening complications. Despite a lack of clear therapeutic recommendations, surgical treatments such as thrombectomy or bypass and/or catheter-directed thrombolysis (CDT) have been first-line procedures in both acute and subacute limb ischemia, but each therapy may lead to significant morbidity and mortality. Such situations demand fast restoration of appropriate flow to preclude limb loss and other complications. Percutaneous mechanical atherectomy plus thrombectomy (MATH) represents a minimally invasive approach for quickly recanalizing thrombus-containing lesions whatever the age of thrombus. Indeed, many chronic patients can present with critical limb ischemia, with thrombus-containing occlusive lesions triggered by underlying atherosclerotic disease. MATH offers various advantages over surgery and CDT, with lower invasiveness, faster recanalization, and the possibility to immediately treat the underlying lesions, with a lower rate of bleeding complications and no need for intensive care unit stay. Currently, several mechanical thrombectomy devices are offered as an alternative therapy and can be divided into pure rotational MATH systems and rheolytic thrombectomy devices. The only pure rotational MATH device currently available on the market is the Rotarex S device. We aimed to review contemporary clinical data regarding the safety, efficacy, and outcomes of MATH therapy using Rotarex S catheter in acute and subacute thrombus-containing arterial lesions of lower limbs. Future perspectives of Rotarex S MATH treatment and cost-effectiveness of its routine use will be also discussed.

## Introduction

Acute and subacute lower limb ischemia is caused by a reduced arterial perfusion to a limb due to either embolic migration or local thrombosis. In addition to the risk of damage to or loss of a limb, it may give rise to life-threatening complications related to systemic metabolic conditions (1). Therefore, in addition to conservative medical measures, treatment will include making a decision regarding the fastest suitable revascularization procedure whatever the underlying setting, native vessel occlusion, in-stent restenosis/occlusion, or bypass thrombosis (2). So far, there are no clear therapy recommendations for acute occlusions. Local thrombolysis is indirectly recommended in stages I and IIa according to the TASC criteria, with mechanical thrombectomy as an alternative (3, 4). In cases of acute, limb-endangering ischemia (stages IIb and III), immediate reperfusion is necessary, usually by surgical approach, but mechanical thrombectomy is also an alternative in this specific situation. Because the results of surgical treatment are often incompletely satisfactory and are associated with higher morbidity and mortality rates, local intra-arterial lysis has prevailed, at least in stages I and IIa, as the standard intervention (3, 4). However, even thrombolysis is associated with systemic complications. It is potentially complex to handle and expensive because patients need to stay in an intensive care unit. Furthermore, a significant bleeding risk, especially in the elderly, can be observed. Endovascular thrombectomy has become an alternative option beside open surgical thrombectomy for the recanalization of thrombotic occlusions in peripheral arterial disease (1, 2). Currently, several mechanical thrombectomy devices, which have the advantage of lower invasiveness, lower risk of complications, and shorter treatment duration, are available as an alternative approach. Various thrombectomy devices are available and may be divided into two different physical action principles: (1) pure rotational mechanical atherectomy plus thrombectomy (MATH) systems and (2) only rheolytic thrombectomy devices (5). The only pure rotational MATH device currently available on the market is the Rotarex S device (Straub Medical, Wangs, Switzerland). The introduction of Rotarex S percutaneous MATH device marks a new era for endovascular treatment and expands the scope for interventional radiologists and vascular specialists.

In this article, we will discuss the current role and future perspectives of MATH therapy using the Rotarex S catheter in acute and subacute thrombus-containing arterial lesions of lower limbs. Future perspectives of Rotarex S MATH treatment and cost-effectiveness of its routine use will be also discussed.

## Technical Aspects of “MATH” With the Rotarex S Catheter

**Figure 1:**
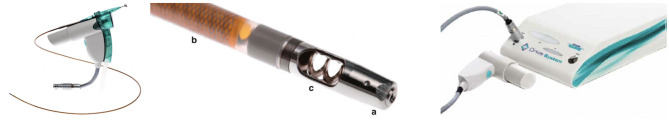
Rotarex S device. **(a)** Cutting head. **(b)** Helix. **(c)** Side window. The handle catheter (*left*) must be connected to the drive system or generator (*right*).

The Straub Rotarex S thrombectomy device works on the Archimedean screw principle (5). The catheter tip is made up of two overlying metal cylinders, with two side openings. The inner cylinder is connected to the catheter shaft and the outer cylinder to the rotating helix. The helix and the catheter tip rotate at ~40,000–60,000 rpm, by means of a gear box in the catheter housing and a motor contained within the catheter handle driven by the Drive System (Figure 1). The outer cylinder is fitted with facets at its foremost head which, when working, serve to abrade thrombotic material lying in front of it. When rotating, both the helix and the outer catheter tip rotate, and are advanced over the guidewire toward an occlusive lesion. When a thrombotic occlusion is met, the rotating head, with its small, blunt facets in its forward aspect, breaks down the material. At the same time, the rotation of the catheter tip creates a vortex effect in the circulation which helps to further erode occlusive material from the vessel lumen. The rotating helix allows to produce a negative pressure inside the device tube to act as a conveyor screw on which the removed material is transported. The detached particles are removed through side windows in the catheter head where they are additionally broken down and removed from the body and into the connected collecting bag under continuous aspiration. It is not necessary for the catheter or rotating tip to be in contact with the vessel wall to be effective. The catheter is designed in such a manner that when used along the guidewire and with appropriate proximal blood flow, no vessel wall damage would result if contact with the wall should unintentionally occur. Different catheter sizes are available: 6, 8, and 10 Fr. Three different lengths are also available depending on the length of the occlusive lesion: 85, 110, and 135 cm. They are composed of three main parts: cutting head, helix, and side window, respectively. The aspiration performance is approximately 0.66 mL/s with the 6-Fr catheter and 1.5 mL/s with the 8-Fr catheter (5, 6).

**Figure 2:**
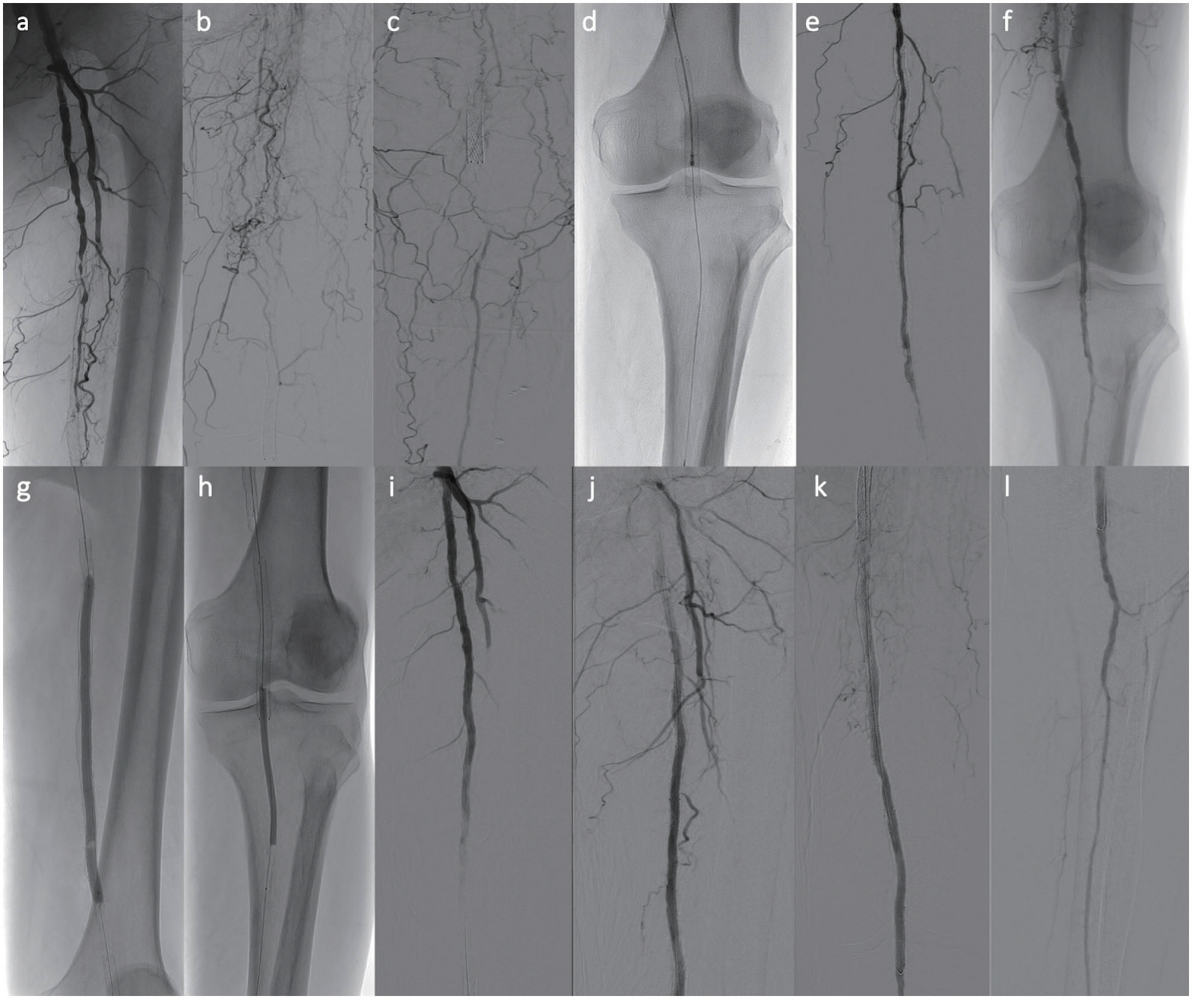
Subacute arterial in-stent thrombotic occlusion in a 69-year-old limping male. **(a–c)** Total in-stent occlusive lesion at the left femoro-popliteal artery level with only one run BTK vessel. **(d)** Use of a 6-Fr Rotarex S catheter over a 0.018-in. micro guidewire for in-stent debulking. **(e,f)** Immediate angiogram after two runs of MATH demonstrating reopening vessel. **(g,h)** Extensive in-stent conventional PTA + DCB angioplasty. **(i–l)** Final angiograms showing excellent results after combined MATH debulking and adjunctive therapy, with normal in-stent flow. No thrombolysis or additional stenting were needed.

After performing a diagnostic arteriogram, a 6-Fr or 8-Fr diameter Rotarex S catheter should be used, depending on the vessel size. Then, using a roadmap, a 0.018-in. micro guidewire is inserted up to the target occlusive lesion and advanced distally beyond it. The Rotarex S device is then advanced along this guide wire up to a few centimeters above the lesion and then activated (Figure 2). It is recommended to pass the occlusion slowly, especially for subacute occlusive lesions with material that is already partially organized to prevent distal embolization. Small forward and backward movements are then performed. Depending on the final result after restoring flow, a percutaneous transluminal angioplasty (PTA) with or without stent placement should be considered, in keeping with the current recommendations. The goal is to prepare the vessel by cleaning up before applying any kind of conventional balloon, drug-coated balloon (DCB), or stent, to respect as much as possible the “leave nothing behind” concept. The catheter is designed either for an antegrade or retrograde (crossover) approach. Frequent flushing of the device outside the patient is mandatory to avoid any dysfunction such as sticking the catheter to the guidewire or breaking the helix.

As antithrombotic regimen, 5,000 IU of heparin is usually injected intraoperatively, followed by low molecular weight heparin treatment for 1 week and 100 mg of acetylsalicylic acid per day as long-term therapy (6).

## Literature Search Strategy

A literature search using the MEDLINE/PubMed, EMBASE, and SCOPUS databases was performed to identify relevant studies published from January 2000 to January 2020. The search terms were “(atherectomy OR thrombectomy OR athero-thrombectomy OR debulking OR Rotarex) AND (acute limb ischemia OR peripheral arterial disease OR chronic limb ischemia OR lower limb occlusion OR leg arteries thrombosis) AND (endovascular treatment OR recanalization) AND (human OR patient).” A few additional studies were found through a manual search of reference lists of other studies and of articles from previous searches. Duplicate publications were identified by juxtaposing author names, study dates, treatment comparisons, sample sizes, or outcomes, and were then excluded.

### Inclusion and Exclusion Criteria

One reviewer performed the literature search and selected the eligible articles.

The selected studies were required to meet the following inclusion criteria: (1) original research article written in English; (2) the study participants were human; (3) prospective and retrospective studies; (4) article presented outcomes of MATH using the Rotarex device for at least five patients.

We excluded studies meeting the following criteria: (1) review articles, letters to the editor, editorials, abstracts, chapters contained within a book, case reports, and preclinical studies; (2) publications that reported data on fewer than five patients; (3) articles presenting no clear results for MATH using the Rotarex device in at least five patients or showing duplicate results.

First, the article titles and abstracts were reviewed. Second, full-text articles were assessed for eligibility.

### Data Extraction and Definition

The following data were recorded from the included full-text articles: first author, publication year, study country, study design (prospective vs. retrospective, comparative or not, randomized or not). For all studies and each arm of comparative studies, the following data regarding the clinical endpoints were extracted: technical success, clinical success, minor and major complications, patency rate, mortality, mean follow-up, and amputation-free survival rate.

## Current Role of “MATH” Using Rotarex S Device in Peripheral Arterial Occlusive Disease

Main data regarding the utility of MATH using the Rotarex S device in the management of acute and subacute lower limb occlusive disease and its potential contribution in prolonging the durability of endovascular revascularization are coming mainly from retrospective studies (Table 1) (10–23). Looking at the literature, we identified 16 clinical studies, which evaluated MATH using the Rotarex S device as a primary treatment during revascularization interventions in patients with peripheral arterial occlusive disease in iliac, femoral, and/or popliteal arteries.

**Table 1:** MATH using the Rotarex S device vs. local thrombolysis vs. surgical thrombectomy for patients with acute limb ischemia: outcomes overview from the main series and trials.

**Author/trial (year)**	**Procedure type**	**Number of patients**	**Clinical success**	**Amputation-free survival within 1 year**
STILE^*^ trial (1994) (7)	Thrombolysis vs. surgery	393	Thrombolysis: 46% Surgery: 74.3%	Thrombolysis: 87% Surgery: 89.6%
TOPAS^*^ trial (1998) (8)	Thrombolysis vs. surgery	544	Thrombolysis: 67.9% Surgery: N/A	Thrombolysis: 65% Surgery: 69.9%
Zeller et al. (2002) (9)	Rotarex	98	97%	95%
Wissgott et al. (2013) (10)	Rotarex	265	94.7%	100%
Stanek et al. (2016) (11)	Rotarex	113	93.8%	90%
Loffroy et al. (2020) (6)	Rotarex S	128	91.4%	93.7%

* *Catheter-directed thrombolysis (STILE: rt-PA or urokinase; TOPAS: urokinase)*.

The majority were retrospective cohorts with a total number of 1,844 patients having had Rotarex S MATH as part of their treatment. Out of 16 studies, 8 studies investigated the role of Rotarex S MATH for native vessel occlusion (12–19), 3 studies used Rotarex S MATH for in-stent restenosis/occlusion (6, 20, 21), 2 studies tested Rotarex S MATH for bypass thrombosis (1, 10), and 3 studies evaluated Rotarex S MATH for mixed populations combining the three previous indications (11, 22, 23).

### Rotarex S “MATH” for Native Vessel Occlusion

Eight retrospective studies reported results regarding the role of Rotarex S MATH in peripheral acute occlusive disease in native vessels, investigating a total number of 1,095 patients (12–19). The largest study was reported by Freitas et al., investigating 525 consecutive patients with a mean age of 66.7 ± 10.7 years (14). Treatment with Rotarex S MATH resulted in significant clinic and hemodynamic improvement in most patients, reducing the need for lytic therapy in a significant proportion of patients. Indeed, Rotarex S MATH was performed solely in 161 (27.2%), Rotarex S MATH plus PTA in 232 (39.1%), stenting in 169 (28.4%), and lysis in 77 (13.9%) procedures only. The procedural technical success rate was 97.7%, with an improvement in Rutherford–Becker category persisting in 74.1% of the patients after 12 ± 2.4 months of mean follow-up. The overall 1-month major adverse events were 6.9%, with a mortality rate of 1.1%. No death was caused by the device. After 1 year, an overall target lesion revascularization (TLR; 10.1%), non-TLR (6.6%), and major amputation rates (2.3%) were reported. Mortality at 12 months was 8%.

Bulvas et al., the largest prospective study, reported 1-year outcomes of Rotarex S MATH as initial therapy in 316 patients (184 men; mean age 70.9 ± 12 years) with acute (202 patients, 63.9%) and subacute (114 patients, 36.1%) lower limb ischemia in native vessel, stent grafts, and bypass grafts (23). Critical limb ischemia was diagnosed in 74 (64.9%) of the 114 patients with subacute limb ischemia. Target occlusive lesions of embolic (*n* = 60) or thrombotic (*n* = 256) origin were visualized in the femoro-popliteal segment (*n* = 231), prosthetic or venous femoro-popliteal bypass grafts (*n* = 75), and the aortoiliac segment level (*n* = 35). The mean occlusive lesion length was 22.9 ± 14.8 cm. Overall technical success rate was 100% after Rotarex S MATH and adjunctive methods (aspiration, PTA, stenting) at the location of the target lesions. No open surgical treatment or lytic therapy was necessary to bypass or recanalize the target arteries. No death was reported associated with target occlusion treatment. Minor complications directly related to the Rotarex S MATH procedure occurred in 26 (8.2%) patients. Major complications occurred in 11 (3.5%) patients. At 1 month, primary and secondary patency rates were 94.3 and 97.2%, respectively, and mortality rate was 0.3%. Amputation-free survival at 12-month follow-up was 87.4%.

In most cases of these studies, the use of Rotarex S MATH was capable of precluding and replacing thrombolysis, and reducing the rate of stenting, showing to be an efficient and safe option for treating acute and subacute thrombus-containing occlusive lesions in lower limb vessels, with excellent immediate and satisfactory long-term outcomes, whatever the age of the thrombus and the type of vessel, even at the proximal iliac level and for long lesions.

### Rotarex S “MATH” for In-stent Restenosis/Occlusion

Three retrospective studies assessed the use of Rotarex S MATH with or without adjunctive therapy (e.g., PTA, DCB, stenting) in the treatment of femoro-popliteal in-stent restenosis or occlusion in a population of 234 patients and showed very encouraging technical success rates ranging from 96.9 to 98.6% (6, 20, 21).

Loffroy et al. reported results of the largest multicenter study in such a setting on 128 patients (6). Overall, critical limb ischemia was observed in 51.5% of patients. At 1-year follow-up, the primary and secondary clinical success rates were 92.3 and 91.4%, respectively. Limb salvage rate was 93.7%. Overall, 32 (25%) reinterventions were reported with a mean time from Rotarex S therapy to reintervention of 7.1 ± 8.2 months. TLR was 19.5% (25/128). Distal embolism occurred in seven (5.5%) patients that responded to endovascular therapy. At mean follow-up, major adverse events were death (18/128, 14.1%), myocardial infarction (9/128, 7.0%), stroke (2/128, 1.6%) and renal failure (3/128, 2.3%).

In the study by Liao et al., the primary patency rate at 1 year was 86.2%, and freedom from clinically driven TLR rate at 1 year was 89.7% (20).

In terms of complications, Milnerowicz et al. reported six (8.1%) critical limb ischemia patients who developed distal embolization that responded to lytic therapy (21). Three (4.1%) dissections did not require treatment, and one (1.4%) perforation required stent-graft implantation. Overall, 33 (44.6%) patients had an additional stent deployed, mainly related to suboptimal outcome or complications. The restenosis rate assessed by Doppler ultrasound scan at 1 year was 20.5%.

Those findings support the use of Rotarex S MATH as primary vessel preparation treatment of in-stent restenosis or occlusion in terms of safety and efficacy. On the other hand, thrombus is highly prevalent in the periphery and quite often under-diagnosed by angiography. Thrombus forms on stents even when not occlusive or angiographically visible. A fine layer of thrombus can affect drug elution into the arterial wall (24). Removing thrombus or modifying its presence may be a promising approach in enhancing drug-eluting balloon/stent technology effectiveness in in-stent restenosis or occlusion.

### Rotarex S “MATH” for Bypass Occlusion

Two retrospective studies including 64 patients, which evaluated Rotarex S MATH for femoro-popliteal bypass thrombosis, showed very promising technical success rates ranging from 82 to 97.6%, whatever the catheter used (1, 10).

Lichtenberg et al. provided data of 22 patients with acute femoro-popliteal bypass occlusion (1). A technical success rate of 82% was shown. During a follow-up period of 6 months, no reinterventions had to be performed. The average ankle brachial index (ABI) after 1 year was 0.80 ± 0.1. One patient showed a hemodynamic restenosis in a Nitinol stent distal to the femoro-popliteal bypass that was implanted during the initial procedure. No reocclusion of the femoro-popliteal bypasses was noted in all patients.

Wissgott et al. investigated the role of rotational MATH using Rotarex S catheter in terms of efficacy and safety in the treatment of acute and subacute femoro-popliteal bypass thrombotic occlusions (10). Forty-two patients (mean age 65.8 ± 9.1 years) were treated. The mean occlusion length was 28.4 ± 2.9 cm. Thirty-four (81%) patients underwent venous bypass, and eight (19%) patients underwent polytetrafluoroethylene bypass. Technical success rate was 97.6%. In one patient, blood flow could not be restored despite the use of the Rotarex S catheter. The average catheter intervention time was 6.9 ± 2.1 min, highlighting the speed of such a procedure. ABI increased from 0.39 ± 0.13 to 0.83 ± 0.11 at discharge and to 0.82 ± 0.17 after 30 days (*P* < 0.05). There were a total of two (4.8 %) peri-interventional complications. One patient developed a distal embolization, which was successfully managed with local thrombolysis, and another patient had a small perforation at the distal anastomosis, which was successfully managed by stenting.

## Rotarex S “MATH” vs. Thrombolysis

There are no randomized studies comparing thrombolysis and Rotarex S MATH. Only indirect comparison is possible. The short- and long-term results of MATH using the Rotarex S device are probably more favorable than those following lytic therapy (3, 4). Especially for old patients and those with comorbidities, it may be significant that Rotarex S MATH, in comparison with lytic treatment, may restore blood flow faster and in one single session (25). No contraindications for Rotarex S MATH exist, in contrast to potentially life-threatening bleeding complications with thrombolysis. Furthermore, there is also no need for hospitalization in intensive care unit after Rotarex S MATH. Hospital stay after Rotarex S MATH is also shorter (23). There is only one exception when lytic therapy cannot be replaced by Rotarex S MATH, in the case of below-the-knee arteries involvement, due to the catheter size.

One single-center retrospective study compared different interventional techniques for the treatment of acute and subacute limb ischemia on thrombotic occlusive lesions of the lower limbs (3). A total of 202 patients, including 26 critically ill patients, underwent Rotarex S MATH, local lytic therapy, or a combination of both. The study showed a primary revascularization success of 98% in all groups. Twelve months after treatment, primary and secondary patency rates after Rotarex S MATH alone were significantly better in comparison with local thrombolysis or a combination of Rotarex S MATH and thrombolysis (63 and 85%, *P* < 0.05). Overall survival 1 year after intervention reached up to 96% in non-critically ill patients, and amputation-free survival was 94.3% in all groups. Mean hospitalization stay and rate of major bleeding complications were significantly increased after lytic therapy compared with Rotarex S MATH (*P* < 0.05).

## Rotarex S “MATH” vs. Surgical Thrombectomy

There are no randomized studies comparing surgical thrombectomy and Rotarex S MATH. Only indirect comparison is possible.

Two old trials compared thrombolysis vs. surgery thrombectomy. The TOPAS study included 544 patients and reported clinical success rates and amputation-free survival after 12 months of 67.9 and 65% in the thrombolysis group (8). Clinical success was not reported in the surgical group whereas amputation-free survival after 12 months was 69.9%. In the STILE study of 393 patients, clinical success rates were 46 and 74.3% in the thrombolysis and surgical groups, respectively (26). Amputation-free survival after 12 months was 87 and 89.6% in the thrombolysis and surgical groups, respectively. In the first five studies reporting results of Rotarex S MATH in 536 patients, clinical success ranged from 93.8 to 100% whereas amputation-free survival after 12 months ranged from 89 to 100%.

Conclusions from these indirect comparisons must be drawn with caution but seem to be in favor of percutaneous MATH in terms of outcomes.

## Rotarex S “MATH” and Cost-Effectiveness

No study exists in the literature focused on the cost of Rotarex S MATH as adjunctive tool in the management of acute and subacute lower limb occlusions, whatever the type of vessels treated. The use of such a catheter obviously leads to additional cost to a revascularization procedure. However, the additional cost is to be balanced with the minimal invasiveness of the intervention, the shorter hospital stay with no need for intensive care unit hospitalization, the faster procedure, the reduced bleeding complication rate, the lower rate of stenting, and the potential lower number of reinterventions, meaning that the use of Rotarex S MATH may be cost-effective when properly used in terms of overall cost on the long-term (9, 27–29). Further studies are warranted.

## Advantages, Drawbacks, and Best Indications of Rotarex S “MATH”

This approach has various advantages: minimally invasive, easy to use, short procedural time, reduced thrombus burden, treats all types of vessels above the knee, low complication rate, no vessel wall damage, successful in restoring vessel patency, unmasks underlying lesion, allows for targeted treatment, no need for lysis drug and vessel preparation, short hospital stay, no intensive care unit stay, and affordable.

On the other hand, the device may have some drawbacks: still kinking problems with ineffective thrombectomy in angled and sclerotic aortic bifurcations, potential danger of vessel wall dissection and perforation in highly calcified vessels, high costs for catheter system, distal embolization still possible with indication for thrombectomy or lysis therapy, no catheter device available for below-the-knee thrombectomy, and minimal lumen vessel 4 mm.

Regarding the best indications, MATH using the Rotarex S device is an effective therapy option for primary re-opening of an infra-aortic vessel, above the knee, whatever the age of lesion. Highly calcified lesions should be avoided because of the risk of perforation. MATH with this system is an efficient treatment alternative for acute or subacute occlusion of a femoro-popliteal bypass as well. Lastly, in-stent restenosis or occlusion with adjunctive therapy probably represents the best indication for MATH use.

## Future Perspectives

Acute and subacute lower limb ischemia represents a real risk not only for the affected limb but also for the entire body. Therefore, fast diagnosis and effective reperfusion strategy are important measures for the patient. In these cases, percutaneous rotational MATH using the Rotarex S device is an effective and safe endovascular therapeutic alternative to the established lysis or surgical therapy (6, 9, 12–15, 26–29). Rotarex S MATH is useful as a vessel preparation tool with the rationale that efficacy depends on the presence of fragmentable and removable occlusive material. The device can be applied for native vessel occlusions, in-stent restenosis/occlusions, or bypass thrombosis. However, although retrospective studies report a high rate of patency and freedom from revascularization, and a low rate of recurrent restenosis with the use of Rotarex S MATH as an adjunctive tool to conventional PTA or DCB application, data from randomized controlled trials are still lacking. Despite a significant improvement in acute technical results and short-term outcomes, wide adoption of this technology during peripheral interventions requires universal algorithm to optimize long-term outcomes. In addition, the next step is to plan a randomized, prospective study with direct face-to-face comparison of interventional percutaneous approach, surgery, and lytic therapy. In addition, studies are needed for evaluation of potential hybrid applications of this technique, in combination with DCB technology with IVUS assessment of residual plaque burden.

Last, even if cost-effective analyses in favor of the adjunctive use of Rotarex S MATH do not exist, this approach allows to decrease the rate of complications in lower limb occlusions (23–25). On the other hand, no data were available on the use of protective filter devices when using the Rotarex S system. Distal embolization rate was quite low, around 5% on average, in the main series reporting results on that as previously described. Further studies are needed to definitely demonstrate the cost-effectiveness of the routine use of Rotarex S MATH during vascular procedures. Indeed, the use of a specific catheter and the expensive equipment which are necessary for the operation of Rotarex S system are the main factors responsible for the increased procedural costs of this technique. However, there is no doubt that Rotarex S MATH treatment is likely to be more often used in the near future for modern endovascular therapy in peripheral arterial occlusive disease.

## Summary and Conclusions

MATH using the Rotarex S device alone or with adjunctive techniques is feasible and safe as a first-line therapeutic option for occlusive lesions in native vessels, stent grafts, and bypasses in patients with both acute and subacute lower limb ischemia. Used as an initial therapy, Rotarex S MATH has a marked potential for reduced morbidity and mortality in comparison with studies of primary lytic or surgical treatment. Despite the issues associated with a randomized clinical study of conventional methods, a well-designed trial of this nature could be able to assess effectively the superior efficacy and safety of Rotarex S MATH therapy compared with lysis or surgery.

Because of the offer of both 6- and 8-Fr devices, the appropriate system for the corresponding thrombus load can be used. Whenever possible, we recommend to use the appropriate size 8-Fr Rotarex S system in the indication of femoro-popliteal bypass occlusion and in-stent restenosis/occlusion to reduce the thrombus load as much as possible and to avoid any additional lysis. Therefore, an effective interventional therapy possibility is available to the affected patient, which avoids open surgery. Especially in patients who are not candidates for an open procedure due to their comorbidities, this technique seems to be a promising interventional alternative.

## Author Contributions

RL, NF, CG, LF, FL, MM, and OC: conception and design of the study, acquisition of data, analysis and interpretation of data, drafting the article, revising it critically for important intellectual content, and Final approval of the version to be submitted. All authors contributed to the article and approved the submitted version.

## Conflict of Interest

The authors declare that the research was conducted in the absence of any commercial or financial relationships that could be construed as a potential conflict of interest.
